# Results of a multi-perspective examination of loneliness trajectories and its determinants in German adults during the COVID-19 pandemic

**DOI:** 10.1038/s41598-025-00678-z

**Published:** 2025-05-23

**Authors:** Anna C. Reinwarth, Mareike Ernst, Denis Gerstorf, Elmar Brähler, Philipp S. Wild, Thomas Münzel, Jochem König, Karl J. Lackner, Norbert Pfeiffer, Manfred E. Beutel

**Affiliations:** 1 Department of Psychiatry and Psychotherapy, University Medical Centre of the Johannes Gutenberg-University Mainz, Mainz, Germany; 2 Department of Psychosomatic Medicine and Psychotherapy, University Medical Centre of the Johannes Gutenberg-University Mainz, Mainz, Germany; 3 Department of Clinical Psychology, Psychotherapy, and Psychoanalysis, Institute of Psychology, University of Klagenfurt, Klagenfurt Am Wörthersee, Austria; 4 Department of Psychology, Humboldt-Universität Zu Berlin, Berlin, Germany; 5 Department of Psychiatry and Psychotherapy, Medical Faculty, University of Leipzig, Leipzig, Germany; 6 Department of Cardiology - Cardiology I, University Medical Centre of the Johannes Gutenberg-University Mainz, Mainz, Germany; 7 Institute of Clinical Chemistry and Laboratory Medicine, University Medical Centre of the Johannes Gutenberg-University Mainz, Mainz, Germany; 8 Department of Ophthalmology, University Medical Centre of the Johannes Gutenberg-University Mainz, Mainz, Germany; 9 Institute of Medical Biostatistics, Epidemiology and Informatics, University Medical Centre of the Johannes Gutenberg-University Mainz, Mainz, Germany; 10 Preventive Cardiology and Preventive Medicine, Department of Cardiology, University Medical Centre of the Johannes Gutenberg-University Mainz, Mainz, Germany; 11 Clinical Epidemiology and Systems Medicine, Centre for Thrombosis and Hemostasis (CTH), University Medical Centre of the Johannes Gutenberg-University Mainz, Mainz, Germany; 12 Institute of Molecular Biology (IMB), Mainz, Germany; 13 German Centre for Cardiovascular Research (DZHK), Partner Site Rhine-Main, Mainz, Germany

**Keywords:** COVID-19 pandemic, Loneliness trajectories, Growth mixture modeling, Protective factors, Risk factors, Cohort study, Risk factors, Public health

## Abstract

Most research on adults’ vulnerability to loneliness during the pandemic has been of limited quality. This study aimed to overcome previous limitations by examining loneliness trajectories in German adults from a population-based cohort during the pandemic using face-to-face assessment, identifying risk factors and highlighting those particularly relevant to older adults. Analyses included two measurement points before and two during the pandemic, combining data from the population-based Gutenberg Health Study and COVID-19 Study (*N* = 7001; baseline: *M*_age_ = 51.72, *SD*_age_ = 10.04). Growth mixture models identified distinct loneliness trajectories. Factors associated with these trajectories were tested by a multinomial logistic regression model including sociodemographic, individual, and pandemic-related predictors and interactions with age. Overall, mean loneliness increased. Three distinct classes were identified: No Loneliness (59.3%), Onset (23.3%), and Temporary Increase (17.4%). In comparison to No Loneliness, Onset was associated with reduction in social contact during the pandemic and Temporary Increase with sex, high school degree, pre-pandemic depression symptoms, pandemic-related stressors and social support. No unique risk factors for older adults were found. Interventions that strengthen one’s adaptability to (acute) stressors and promote social resources with special attention to women may be a promising way to prevent loneliness during the pandemic.

## Introduction

The COVID-19 pandemic has impacted the lives of many people. Public health measures of “social distancing” to slow down the spread of the virus have increased the risk of loneliness^1^^.^

Even before the pandemic, loneliness has come to the fore of public health and political concerns in today’s aging society due to its major impact on physical and mental health^2–4^. Loneliness and its associations with health and well-being have previously been investigated from different perspectives^5^. The cognitive discrepancy theory, one of the most influential theoretical approaches to loneliness, defines loneliness as an unpleasant, subjective experience arising from a discrepancy between individuals’ desired and actual social relations, either quantitatively or qualitatively^6^. By conceptualizing loneliness upon a cognitive approach, the discrepancy theory provides explanations for feelings of loneliness despite having a multitude of social relationships and for the absence of loneliness in situations of objectively low social integration^7^. It offers the opportunity for a comprehensive understanding of the complex phenomenon of loneliness and its variability. Due to the subjective nature of loneliness, it differentiates itself from objective constructs such as social isolation that is based on the quantity of social relationships and describes the externally visible situation^8,9^. The theory suggested further, that the discrepancy may be caused by changes in one’s social relations and/or changes in one’s social needs or desires^6^. For instance, previous research has attested to changes in loneliness associated with major life events such as marriage or separation^10^. It could be argued that the pandemic also has brought about pervasive changes in social relations due to restrictions on physical social interaction. Spontaneous contact at the workplace decreased, and local, community-level interactions (e.g., with neighbours) became more critical. Also, not just work meetings but also informal exchanges shifted to the digital sphere^11^. At the same time, research suggests changes in the subjective perception during the pandemic, e.g., the importance of social interactions^12^. Moreover, times of stress or uncertainty affected the person´s desire of affiliation^13^. The pandemic represents a unique multidimensional stressor^14^ and has provided a source of mental distress^15–17^, that may lead to changes of desired social relations.

Moreover, the discrepancy theory takes into account several individual predisposing factors that further increase one’s vulnerability, e.g., sociodemographic characteristics^18^, a person’s living situation^19^, their personality^20^, social expectations and cultural norms^21,22^.

A comprehensive recent systematic review with meta-analysis of longitudinal studies with pre-pandemic comparisons has shown global loneliness increases since the pandemic’s start^1^. However, the pooled effect was small (Standardized Mean Difference, SMD = 0.27) and heterogeneous (95% confidence interval, 95% CI = 0.14–0.40), highlighting that the pandemic did not affect everyone similarly. Specifically, female sex, age above 64 years, low education or financial resources, living alone or in urban areas, and pre-existing mental disorders were reported as risk factors for loneliness during the pandemic^23–26^. Along these lines, older adults, a subgroup of adults aged 65 years and older^27^, have been considered as a risk group even before the pandemic^28^. From 2021–2023 one in five older adults was affected by loneliness^29^. Older adults’ risk for loneliness might increase during the pandemic for several reasons. Strict adherence to distancing were critical for older adults due to their higher risk of severe disease courses if infected. Furthermore, many older adults lacked access and/or skills to use online communication channels that could have relieved loneliness^30^. However, results are still inconsistent regarding loneliness among older adults during the pandemic. Several studies, reported increases in mean loneliness during the pandemic^31–33^, whereas others found more stable loneliness levels^34,35^.

Nevertheless, as recent studies mainly focused on mean changes, the heterogeneity among adults and especially older adults might have been disregarded. Only a few studies have identified trajectories of loneliness, ranging from resilience/low loneliness to increasing/high loneliness during the pandemic in non-German adults^25,36^. However, the representativity of these is questionable. Many studies were limited by convenience samples^37^ or running online/switching to an online format^32^.

There is a paucity of data from German prospective, population-based, large-scale cohort studies with face-to-face assessments. To devise targeted prevention and intervention efforts, it is crucial to identify those adults most susceptible to loneliness during the pandemic with the accompanying pre-existing as well as pandemic-related factors. Going from there, in contrast to the plethora of studies coming out showing a cross-sectional snapshot of loneliness during the pandemic, using convenience samples or collecting data online, the present study contributes to the recent literature by examining loneliness trajectories and its determinants based on a German population-based, longitudinal cohort study including face-to-face assessments of four measurement points across 2007–2021. As older adults may be a vulnerable group during the pandemic, the present study also tests interaction effects with age. Specifically, the present study aimed to examine distinct loneliness trajectories among German adults, to identify sociodemographic, individual, and pandemic-related factors associated with the loneliness trajectories by examining the role of pre-existing vulnerabilities and pandemic-related resources and stressors, and to highlight associations characteristic of older adults in an exploratory approach.

## Results

Data of *N* = 7,001 participants with a mean age of 51.72 years (*SD* = 10.04; range = 35–74 years) at T1 were analysed. The sample included 48.9% women and 51.1% men. Table 1 reports detailed sample characteristics.

**Table 1:** Participant characteristics (N = 7001 at baseline) (Gutenberg Health Study and Gutenberg COVID-19 Study, Germany, 2007–2021).

Sociodemographic factors	
Sex (N, %)	
Men	3575 (51.06)
Women	3426 (48.94)
Age (*M*, *SD*)	51.72 (10.04)
High school degree (*N*, %)	
Yes	3283 (46.89)
No	3708 (52.96)
Occupational status (*N*, %)	
Employed	5202 (74,30)
Unemployed	106 (1.51)
Retired	1295 (18.50)
Other	390 (5.57)
Equivalized income (median, IQR)	2.58 (1.60)
Partnership (*N*, %)	
No	1686 (24.08)
Yes	5315 (75.92)
Living alone (*N*, %)	
No	6143 (87.74)
Yes	858 (12.26)
Loneliness and social factors	
Pre-pandemic loneliness (*M*, *SD*)	1.15 (0.51)
Social support (*M*, *SD*)	20.25 (4.22)
Individual differences	
Resilient coping (*M*, *SD*)	10.45 (3.44)
Physical health	
Pre-existing physical illness (*N*, %)	
No	2876 (41.08)
Yes	4048 (57.82)
Mental distress	
Pre-pandemic depression symptoms (*M*, *SD*)	3.95 (3.43)
Pre-pandemic anxiety symptoms (*M*, *SD*)	0.85 (1.10)
Pre-pandemic social phobia symptoms (*M*, *SD*)	2.29 (2.02)
Pandemic-related factors (*M*, *SD*)	
Resources	15.16 (4.26)
Stressors	20.28 (5.55)
Difference in social contact before- versus during-pandemic	2.58 (3.09)

Participant characteristics are shown as mean values and standard deviations or as percentages and absolute numbers; reported percentages related to the total sample (*N* = 7001); occupational status “others” summarizes maternity leave, homemaker, in training or part-time retirement; equivalized income is shown in units of 1,000€ and described by the median with interquartile range; Pre-existing physical illness includes one of the following diagnoses at baseline: hypertension, diabetes, cancer, COPD, CAD, dyslipidemia; Pre-pandemic loneliness was measured by the single item “I am frequently alone/have few contacts”; Pre-pandemic depression symptoms were measured by the PHQ-9; Pre-pandemic anxiety symptoms were measured by the GAD-2; Pre-pandemic social phobia symptoms were measured by the mini-SPIN; Social support was measured by the BS-6; Resilient coping is measured by the BRCS; *M* = Mean, *SD* = Standard deviation.

### Brief summary of the key findings

During the pandemic, average levels of loneliness increased significantly. We identified three distinct loneliness trajectories: 59.3% of participants were classified as not lonely, 23.3% of participants showed an increase in loneliness, and 17.4% of participants reported a temporary increase in loneliness during the study period. Participants who became lonely reported a large reduction in social contact during the pandemic, while those who felt temporarily lonely were more likely to be female, have a high school education, have more pre-pandemic depression symptoms, pandemic-related stressors and low social support than non-lonely participants.

### Descriptive analyses of loneliness

Mean levels of loneliness increased significantly over the four measurement points (*M*_*T1*_ = 1.15, *SD*_*T1*_ = 0.51; *M*_*T2*_ = 1.16, *SD*_*T2*_ = 0.52;* M*_*T3*_ = 1.52, *SD*_*T3*_ = 0.82; MT_*4*_ = 1.83, *SD*_*T4*_ = 0.95, *p* < 0.01).

### Identification of distinct loneliness trajectories

The three-class model for loneliness was considered the best fit showing the lowest BIC (39,582.03) and SABIC (39,528.01), the second highest entropy (0.7825253), and still a significant improvement from the two classes model in log-likelihood (VLMR-LRT = 3787.760; *p* < 0.001). Even though the four-class model had the highest entropy (0.7932044), the VLMR-LRT showed no significant improvement from the three-class- model to the four-class model (− 521.735; *p* = 1.000). Therefore, we opted for the three-class-model (for details, see Supplementary Table 1):

- Class 1: No Loneliness (59.3% of participants): This class showed no loneliness before and during the pandemic.
- Class 2: Onset of Loneliness (23.3% of participants): This class showed an increase from no/slight loneliness to higher values at the first during-pandemic measurement point (T3) with moderate overall levels at the second during-pandemic measurement point (T4).
- Class 3: Temporary Increase in Loneliness (17.4% of participants): This class showed an increase from slight loneliness before the pandemic (T1, T2) to moderate loneliness at the first during-pandemic measurement point (T3) and attenuation at the second during-pandemic measurement point, achieving pre-pandemic loneliness levels (T4).

**Fig. 1:**
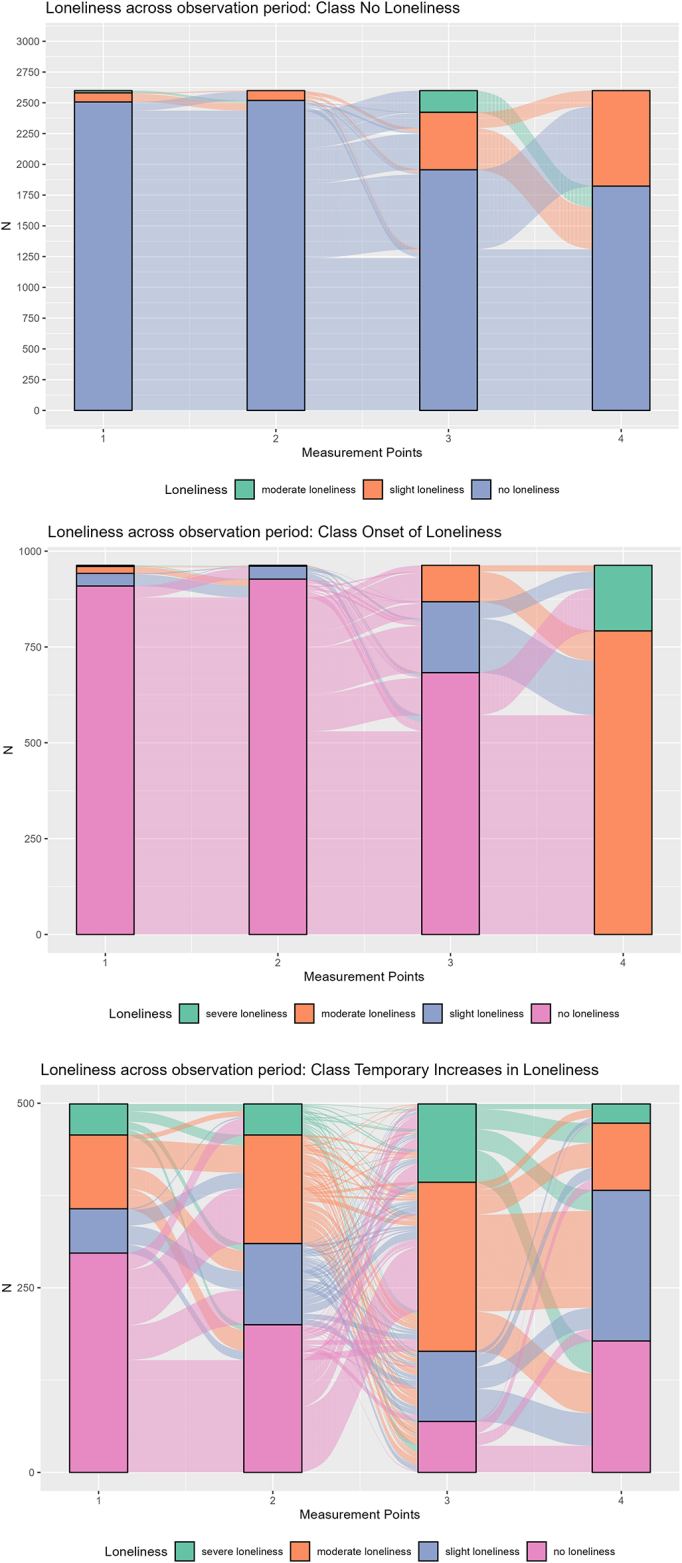
(**a**–**c**) Number of lonely participants within the three latent classes for all times of measurement: (**a**) = No Loneliness; (**b**) = Onset of Loneliness; (**c**) = Temporary Increases in Loneliness. The figures only show participants with available data at all four measurement points. *N*_No Loneliness_ = 2,599; *N*_Onset of Loneliness_ = 963; *N*_Temporary Increase in Loneliness_ = 499

The three classes’ levels of loneliness at all measurement points are shown in Fig. 1a–c.

### Factors associated with latent class membership

Table 2 shows the multinomial logistic regression results exploring the factors associated with class membership of Onset of/Temporary Increase in Loneliness compared to No Loneliness. The model comprised sociodemographic, individual, and pandemic-related factors and these domains’ interactions with age.

**Table 2:** Multinomial logistic regression analyses for latent class membership concerning changes in loneliness in interaction with age in the full sample (Gutenberg Health Study and Gutenberg COVID-19 Study, Germany, 2007-2021)

Variable	Onset of loneliness			Temporary increase in loneliness		
	Odds ratio	95%-confidence interval	*p*	Odds ratio	95%-confidence interval	***p***
Sociodemographic factors						
Age group (Ref.: < 65 years)	1.07	0.97–1.18	0.16	0.97	0.86–1.09	0.58
Sex (Ref.: Men)	0.92	0.74–1.15	0.47	1.48	1.14–1.94	** < 0.01**
Sex x age group	0.93	0.60–1.43	0.74	1.00	0.57–1.75	0.99
High school degree (yes)	1.26	1.00–1.58	0.05	1.62	1.24–2.12	** < 0.01**
High school degree (yes) x age group	0.66	0.43–1.02	0.06	1.00	0.56–1.81	1.00
Occupational status						
Retired (yes)	0.36	0.04–3.08	0.35	0.47	0.08–2.69	0.39
Occupational status x age group	2.04	0.23 -17.96	0.05	2.26	0.36 -14.10	0.38
Equivalized income	0.99	0.94–1.05	0.78	1.00	0.93–1.07	0.91
Equivalized income x age group	1.03	0.92–1.14	0.62	0.90	0.74–1.09	0.29
Partnership (yes)	1.04	0.77–1.42	0.79	0.74	0.53–1.03	0.08
Partnership x age group	0.76	0.40–1.46	0.45	1.07	0.46–2.52	0.87
Living alone (yes)	1.24	0.81–1.91	0.32	1.45	0.93–2.25	0.10
Living alone x age group	0.66	0.28–1.55	0.35	0.61	0.21–1.72	0.35
Loneliness and social factors						
Pre-pandemic loneliness	0.64	0.50–0.81	** < 0.01**	0.74	0.57–0.96	0.02
Pre-pandemic loneliness x age group	1.32	0.89–1.96	0.16	0.87	0.54–1.41	0.58
Social support	1.01	0.97–1.04	0.71	0.91	0.88–0.94	** < 0.01**
Social support x age group	0.98	0.93–1.03	0.38	1.00	0.94–1.06	1.00
Individual differences						
Resilient coping	1.02	0.98–1.06	0.26	0.96	0.92–1.00	0.08
Resilient coping x age group	0.96	0.91–1.02	0.20	0.97	0.90–1.05	0.52
Physical health						
Pre-existing physical illnesses (yes)	0.82	0.65–1.02	0.08	0.80	0.61–1.05	0.11
Pre-existing physical illnesses x age group	1.85	1.16–2.95	0.01	1.04	0.58–1.89	0.89
Mental distress						
Pre-pandemic depression symptoms	1.01	0.96–1.06	0.69	1.13	1.08–1.19	** < 0.01**
Pre-pandemic depression symptoms x age group	0.96	0.88–1.05	0.41	0.96	0.87–1.06	0.41
Pre-pandemic anxiety symptoms	0.98	0.86–1.11	0.72	0.96	0.84–1.11	0.60
Pre-pandemic anxiety symptoms x age group	1.23	0.95–1.60	0.12	1.32	0.98–1.78	0.07
Pre-pandemic social phobia symptoms	1.04	0.97–1.04	0.20	0.99	0.93–1.06	0.73
Pre-pandemic social phobia symptoms x age group	0.95	0.85–1.07	0.34	1.09	0.95–1.25	0.22
Pandemic-related factors						
Resources	1.00	0.97–1.02	0.74	0.96	0.93–0.99	0.02
Resources x age group	1.00	0.96–1.05	0.81	0.98	0.92–1.05	0.64
Stressors	1.02	0.99–1.04	0.15	1.11	1.08–1.13	** < 0.01**
Stressors x age group	1.01	0.97–1.05	0.66	1.06	1.00–1.11	0.03
Difference in social contact before- versus during-pandemic	1.10	1.07–1.14	** < 0.01**	1.03	0.99–1.07	0.17
Difference in social contact before- versus during-pandemic x age group	0.95	0.89–1.01	0.11	0.94	0.86–1.02	0.16

*N* = 2870; the reference category is the no loneliness class; equivalized income is shown in units of 1000€ and described by the median with interquartile range; pre-existing physical illness includes one of the following diagnoses at baseline: hypertension, diabetes, cancer, COPD, CAD, dyslipidemia; Pre-pandemic loneliness was measured by the single-item “I am frequently alone/have few contacts”; Pre-pandemic depression symptoms were measured by the PHQ-9; Pre-pandemic anxiety symptoms were measured by the GAD-2; pre-pandemic social phobia symptoms were measured by the mini-SPIN; Social support was measured by the BS-6; Resilient coping was measured by the BRCS; Pandemic-related factors (resources and stressors) were included as the sum score; Difference in social contact before and during pandemic was included as the continuous score; pseudo-R^2^ values: Cox and Snell = .17 , Nagelkerke = .20, McFadden = .10.

Significant values are in bold.

#### No loneliness versus onset of loneliness

A larger reduction in social contact before vs. during the pandemic was associated with increased odds of Onset of Loneliness [OR (95% CI) = 1.10 (1.07–1.14), *p* < 0.01]. Higher levels of pre-pandemic loneliness was associated with reduced odds of Onset of Loneliness [OR (95% CI) = 0.64 (0.50–0.81), *p* < 0.01]. No interaction effect with age was observed.

#### No loneliness versus temporary increase in loneliness

Female sex [OR (95% CI) = 1.48 (1.14–1.94), *p* < 0.01], having a high school degree [OR (95% CI) = 1.62 (1.24–2.12), *p* < 0.01], more pre-pandemic depression symptoms [OR (95% CI) = 1.13 (1.08–1.19), *p* < 0.01] and more pandemic-related stressors [OR (95% CI) = 1.11 (1.08–1.13), *p* < 0.01] were associated with increased odds of Temporary Increase in Loneliness. In contrast, more social support [OR (95% CI) = 0.91 (0.88–0.94), *p* < 0.01] was associated with reduced odds of being part of the Temporary Increase in Loneliness class. There was no interaction with age.

## Discussion

The present study aimed to identify distinct loneliness trajectories among German adults and associated risk/protective factors against the backdrop of the pandemic. Towards this aim, growth mixture models followed by multinomial logistic regression analyses, drawing on comprehensive data on sociodemographic, individual, and pandemic-related factors were used. In addition, we have tried to highlight predictors that are characteristic of older adults aged 65–88 years.

Reproducing previous findings^1^, descriptive analyses showed an increase in the mean level of loneliness since the onset of the pandemic. Growth mixture models indicated three latent classes of loneliness trajectories. Most participants reported no loneliness before and during the pandemic. About one in four participants reported an onset of loneliness after the initial phase of the pandemic. The remaining just under one-fifth had a more volatile profile, with a temporary increase in loneliness in the initial phase of the pandemic followed by a decrease. The findings mirror previous studies analysing loneliness trajectories in adulthood during the pandemic, with most individuals not being lonely^36,37^. However, the present and previous studies also highlighted vulnerable subgroups experiencing an onset or increase of loneliness, suggesting that about a third of adults are at risk for detrimental trajectories.

Based on the theoretical implications of the cognitive discrepancy theory^6^, it could be argued that the pandemic and accompanying measures of social distancing have led to a mismatch between desired and actual social relations increasing the risk of loneliness. Limitations of physical social contact might have reduced social interactions and in turn affected the extent of actual social relations. Additionally, worries about possible infection or loss of loved ones might have act as pandemic-related stressors and increased needs of social support during pandemic^32^. The distinct loneliness trajectories of the two vulnerable subgroups may reflect different processes or drivers of changes in social relations and needs: If loneliness arises from a discrepancy between one’s social needs and actual social relations, either quantitatively or qualitatively, one could hypothesize that loneliness in the initial phase of the pandemic might be primarily driven by quantitative changes in relationships in the wake of restrictions on physical contact, as shown by the loneliness-aggravating effects of reductions in social contacts during the pandemic in this study. Over time, individuals might have compensated these initial changes by increased use of digital communication and social interaction channels leading to attenuation of feelings of loneliness in later stages of the pandemic. Furthermore, one could also hypothesize a cushioning effect of newly available pandemic-related resources (e.g., more time with family, strong public support and cooperation to face the shared public health threat) has compensate the reduction of social interactions in individuals who mentioned a temporary increase^38^. Whereas individuals who suffered from an onset might have failed to find ways of compensation changes in social relations over time.

Further risk/protective factors also mirrored previous patterns^25,37^, with an increased vulnerability conferring by female sex, socioeconomic and social disparities. Higher burden of pre-pandemic depression symptoms and pandemic-related stressors differentiated further between the No Loneliness and the Temporary Increase class, similar to previous findings^36^. Also, comparable to earlier findings, social support was a protective factor^25,36^. Higher pre-pandemic loneliness was related to a lower risk of loneliness onset during the pandemic, most likely indicating a ceiling effect.

No interaction effects of the included predictors with age were found indicating similar factors shaping one’s vulnerability to loneliness in old age.

A strength of the present study was the longitudinal study design with the availability of repeated measurements from a representative, large-scale cohort study with a prospective design and unchanged, high-quality methodology before and during the pandemic. The extensive examination at the study centre allowed for testing a wide range of differences in sociodemographic, individual, and pandemic-related factors within participants with a substantial age span.

The limitations of the study in terms of representativeness, measurement issues and study period need to be taken into account when interpreting the results.

As the present findings were based on data from the German general population, it is still being determined to what extent these can be transferred to other countries, particularly middle- and lower-income countries. Moreover, as study participation required on-site visits, older adults who lived in, or moved to nursing homes, respectively, and those who were house-bound were most likely underrepresented. As our sample only included adults up to the age of 88, our findings cannot be generalised to older adults in their late 80 s and 90 s, who may be particularly vulnerable to loneliness due to increased mobility and health, and lower levels of technological literacy, which further limit social connectedness. While data of 7001 participants at T1 and at T3 were available, at T2 and T4 6906 and 6518 participants provided data, respectively. This attrition may have affected our results, as those who withdrew may be lonelier than those who participated at all four measurement points.

A unidimensional single item measured loneliness. Therefore, we cannot investigate different types of loneliness or their separate trajectories as previous studies did, e.g., van der Velden et al.^37^ reported no difference in the prevalence of ‘social and emotional loneliness’, while ‘emotional loneliness’ alone had increased. Moreover, in addition to social and emotional loneliness Landmann and Rohmann^39^ showed that physical loneliness (perceived lack of physical contact) constitutes a third type, especially relevant in times of social distancing during the pandemic. The three types were found to differ in their prevalence during the pandemic and in their associations with age and personality traits. While emotional and social loneliness remained stable, physical loneliness increased during the contact restrictions. We concede, that the used single-item may only capture the physical typ. By asking quite explicitly whether a person was often alone and suffered from it, the results might also have been affected by biases such as social desirability or the perceived stigmatization of loneliness. As the single-item is a double-barred statement by combining frequently alone and few contacts, we cannot be certain which part of the item the participants were responding to.

In addition, the two item blocks on resources and stressors related to the pandemic were developed by the research team specifically for the GCS. Although the research team drew on previous German studies^40,41^ analysing the impact of the pandemic in formulating the items, the items were not piloted or reliability tested. We therefore have no information about their validity.

Lastly, the present study’s most recent measurement point occurred between March and June 2021 when the pandemic was still ongoing, hindering predictions about further developments and the stability of the last-observed loneliness levels in the distinct trajectories which would necessitate follow-ups.

Summed up, a majority of adults were not lonely. Two sizeable, distinct subgroups reported at least temporary loneliness. Findings give new insights into the variability of its temporal dynamic. Considering the substantial variability in loneliness, we propose that future research continues monitoring the pandemic’s medium- and long-term impact.

Moreover, we showed that loneliness depends on many influencing factors, e.g., sociodemographic disparities, pre-existing vulnerabilities, pandemic-related factors and social resources.

Consistent with recent research^42–44^, our findings show that perceived social support plays an important role in strengthening adaptability and resilience to (acute) stressors. Accordingly, the promotion of perceived social support is an important component in the development of prevention and intervention programmes to strengthen resilience in the context of loneliness as a psychosocial consequence of the pandemic. Taking into account the protective effect of perceived social support, it is recommended, in the sense of social facilitation^45^, to offer as many (digital) contact opportunities as possible in the form of social evenings in churches or retirement homes as well as sports or art groups in order to minimise the negative psychosocial consequences of the pandemic. However, it should be borne in mind that simply creating opportunities for contact will not reach all lonely people. In particular, people affected by loneliness rarely take up such offers^46^. Here, a support person can motivate people to make contact^47^. Further, prevention and intervention strategies may be focused on one’ attitudes, beliefs, and behaviour. Improving quality and perception of social support by social skills, management of maladaptive attributional biases or symptoms of social anxiety may represent a promising approach^48,49^.

Nevertheless, as loneliness is a multidimensional and subjective phenomenon, it is important to remember that there is no one-size-fits-all approach that will work for all adults. Prevention and intervention programmes are likely to be most effective if they are adaptable to individual and societal changes. Thus, researchers, health care systems and political decision-makers should encourage to update the knowledge about risk/protective factors regularly, taking different domains (e.g., individual, actual situation, society) into account.

## Methods

### Study design and participants

Data were drawn from the ongoing, prospective Gutenberg Health Study (GHS) and its pandemic-specific add-on study, the Gutenberg COVID-19 Study (GCS). The GHS started in 2007 in the Rhine-Main region in western Mid-Germany^50^.

The GHS sample was drawn randomly from the local registries of the city of Mainz and the district of Mainz-Bingen, stratified 1:1 for sex and residence and in equal strata across age decades. Individuals aged 35 to 74 at baseline were eligible to participate. Insufficient knowledge of the German language and physical or mental inability to visit the study centre for investigation were exclusion criteria. During an extensive 5-h examination in the study centre, performed by certified medical technical assistants following standard operating procedures, cardiovascular risk factors and other clinical variables were assessed, complemented by a computer-assisted personal interview, laboratory examinations from venous blood samples, blood pressure, and anthropometric measurements. The local data safety commissioner and the ethics committee of the Medical Chamber of Rhineland-Palatinate approved the protocol and documents of the study (reference no. 837.020.07; original vote: March 22, 2007, latest update: December 20, 2020). All study investigations were conducted in line with the Declaration of Helsinki and principles outlined in recommendations for Good Clinical Practice and Good Epidemiological Practice. Written informed consent was obtained from all participants, before inclusion in the study.

**Fig. 2:**
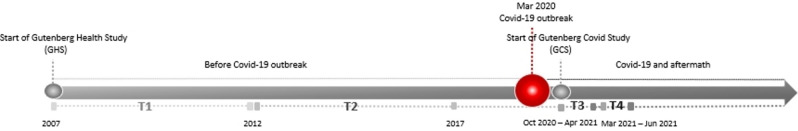
Visualization of the four measurement points of the present study. The Gutenberg Health study (GHS) is a prospective, ongoing community cohort running since 2007. The Gutenberg Covid Study (GCS) is an add-on project within the GHS starting shortly after the Covid-19 outbreak to better understand the challenges associated with the pandemic.

The study comprised data from four waves of measurement: The GHS baseline assessment (T1; first measurement point; 2007–2012), the GHS five-year follow-up (T2; second measurement point; 2012–2017), the GCS baseline assessment (T3; third measurement point; October 2020–April 2021) and the GCS four-month follow-up (T4; fourth measurement point; March–June 2021). Figure 2 provides an overview of the study design.

### Measures

Sociodemographic factors were assessed via self-report and included sex (male/female), age in years, high school degree (yes/no), occupational status (employed, unemployed, retired, other), partnership (yes/no) and living alone (yes/no). In line with previous research, we calculated the equivalized income per month in euros by dividing a household’s total monthly net income by a weighted household size. The first adult was weighed by a factor of 1.0, every additional household member ≥ 14 year was weighed by adding a factor of 0.5, and every child < 14 year was weighed by adding a factor of 0,3 to the weighing scale^51^.

#### Loneliness and social factors

Loneliness was measured by a validated single item. Participants responded to the statement “I am frequently alone/have few contacts”. Response options were 0 (“no, does not apply”), 1 (“yes, it applies, but I do not suffer from it”), 2 (“yes, it applies, and I suffer slightly”), 3 (“yes, it applies, and I suffer moderately”), or 4 (“yes, it applies, and I suffer strongly”). Loneliness was recoded combining 0 and 1 to indicate "no loneliness or distress", 2 = “slight”, 3 = “moderate”, and 4 = “severe loneliness” in line with previous research^52^. Values ≥ 2 indicated “loneliness”. The single-item was previously developed for the use in the GHS^53^. To date, it is validated and widely used in psychological research^52–55^. Comparisons with the established German Version of the UCLA Three-item loneliness scale, the loneliness scale-SOEP (LS-S)^56^. indicated similar prevalence rates of loneliness on both measures, supporting its validity^52^. Further, its validity has been demonstrated by relations to depression and anxiety symptoms, satisfaction with life, household size, and partnership^52–54^. Overall, findings showed that the single-item can be used for screening efforts of loneliness within large-scale surveys when situational constraints limit the use of longer scales (e.g., limited time and financial resources)^52^.

Social support was determined using the Brief Social Support Scale (BS-6)^19^. The BS-6 consists of six items rated on a 4-point Likert scale from 0 (“never”) to 3 (“always”), covering the availability of emotional-informational and tangible social support, e.g., “If you needed it, how often is someone available to take you to the doctor if you need it; to give you good advice about a crisis?”. Answers were summarized to a sum score (0–18). Higher scores indicated higher social support. In the present sample, the BS-6 showed good internal consistency (ω = 0.94).

#### Individual differences

The Brief Resilience Coping Scale (BRCS)^57^ was used to assess resilient coping by inquiring about respondents’ ability e.g., to alter difficult situations or to adjust/control their reactions. Answers are rated on a 5-point Likert scale from 0 (“does not apply at all”) to 4 (“does apply fully”) and summarized to a sum score (0–16). Higher scores indicated greater resilient coping. In the present sample, the BRCS showed good internal consistency (ω = 0.83).

#### Physical health

Pre-existing physical illnesses were operationalized as a binary variable about the absence of all and the presence of one of the following medical diagnoses at baseline: hypertension, diabetes, cancer, chronic obstructive pulmonary disease, coronary artery disease, or dyslipidemia, respectively.

#### Mental distress

The depression module of the Patient Health Questionnaire (PHQ-9)^58^ was used to assess depression symptoms. Participants indicated e.g., loss of interest or loss of/increased appetite on a four-point scale from 0 (“not at all”) to 3 (“nearly every day”) concerning the last two weeks. Responses were summarized to a sum score (0–27). Higher scores indicated higher levels of symptomatology. In the present sample, the PHQ-9 showed good internal consistency (ω = 0.83).

Anxiety symptoms were measured using the Generalized Anxiety Disorder Screener (GAD-2) ^59^. On a four-point scale from 0 (“not at all”) to 3 (“nearly every day”), participants rated to what extent they were affected by the feeling of nervousness, anxiety, and the inability to stop/control their worrying over the last two weeks. Answers were added to a sum score (0–6). Higher scores indicated higher levels of symptomatology. Results of a large population-based study showed acceptable internal consistency (α = 0.82)^59^.

The short form of the Social Phobia Inventory (mini-SPIN)^60^ was used to assess social phobia symptoms, capturing e.g., avoiding doing things/speaking to people or fear of being in the centre of attention. The five-point Likert scale ranges from 0 (“not at all”) to 4 (“extremely”). Answers were summarized to a score (0–12). Higher scores indicated higher levels of symptomatology. In the present sample, the mini-SPIN showed an acceptable internal consistency (ω = 0.78).

#### Pandemic-related resources and stressors

Pandemic-related resources were rated in a block of seven single items (e.g., more time with family or higher appreciation) on a 4-point Likert scale from 1 (“does not apply at all”) to 4 (“applies completely”). Answers were summarized to a score (1–28). Higher scores indicated more pandemic-related resources. Pandemic-related stressors were rated in a block of ten single items. Participants were asked if and how much they suffered from several pandemic-related burdens (e.g., fear of infection or loss of social contacts). Response options were 0 (“does not apply”), 1 (“not at all burdensome”), 2 (“slight”), 3 (“moderate”), 4 (“fairly”) and 5 (“extremely burdensome”). Stressors were recoded by combining 0 and 1 to indicate 1 = “no burdens”, 2 = “slight”, 3 = “moderate” and combining 4 and 5 to indicate 4 = “severe burdens”. Answers were summarized to a sum score (1–40). Higher scores indicating more pandemic-related stressors. The two item-blocks were developed specifically for the GCS by the research team. In formulating the items, the research team drew on SOEP Core^41^ and DynaCORE^40^, German studies that also analysed the impact of the pandemic on daily life and mental health.

Before and during the pandemic, social contact was captured by six single items on the kind and frequency of face-to-face and digital social contact developed specifically for the GCS. The response options ranged from 0 (“never”) to 5 (“daily”). Answers were summarized to a score (0–30). Higher scores indicated more frequently social contact. The difference in social contact before and during the pandemic was calculated by subtracting the during-pandemic from the before-pandemic score.

### Statistical analysis

Descriptive characteristics of the sample are reported as absolute numbers/percentages for categorical variables and as means with standard deviations for continuous variables.

Growth mixture models were constructed to identify loneliness trajectories with linear and squared effects for the time, parameterized as time since T1, and these estimated fixed and random effects for intercepts and slopes. Models with one to four classes (because models with more classes no longer converged in a reasonable time) were fitted using the four repeated measures of loneliness. Each model with two or more classes was run multiple times with varying random start values (100 iterations) to avoid the model identifying local maxima. The Bayesian information criterion (BIC), the sample size-adjusted Bayesian information criterion (SABIC), the adjusted Vuong-Lo-Mendell- Rubin likelihood ratio test (VLMR-LRT), and a measure of entropy were used to determine model fit. After identifying the model with the optimal number of classes, associations between class membership and sociodemographic, individual, and pandemic-related factors were tested in a multinomial logistic regression model including the predictors’ interactions with age. To identify factors that are specifically relevant for older adults, age was dichotomised into two age groups of < 65 versus ≥ 65 based on the age at T3. The no loneliness class was the reference category in all multinomial logistic regression models. The coefficients (logged odds) of the model were converted into interpretable odds ratios by exponential analyses. *P*-values were calculated using Wald tests. A factor was considered to be of significance with a two-tailed value of *p* < 0.01. The goodness of fit of the multinomial logistic regression model was determined by Pseudo-R^2^ statistics (Cox and Snell, Nagelkerke, McFadden). All calculations were performed using the statistical program R (version 4.2.1, packages: lcmm, tidyLPA, haven, reshape2, dplyr, rlang, naniar, psych, fastdummies, nnet).

## Data Availability

**T**he datasets analysed during the current study are not publicly available. The written informed consent of the study participants is not suitable for public access of the data and this concept was not approved by the local data protection officer and ethics committee. But access to data at the local database in accordance with the ethics vote is offered upon reasonable request at any time. Interested researchers make their requests of the Principal Investigator of the GHS (Philipp.Wild@unimedizin-mainz.de).

## References

[1] Ernst, M. et al. Loneliness before and during the COVID-19 pandemic: A systematic review with meta-analysis. *Am. Psychol.*10.1037/amp0001005 (2022). 35533109 10.1037/amp0001005 PMC9768682

[2] Holt-Lunstad, J., Smith, T. B., Baker, M., Harris, T. & Stephenson, D. Loneliness and social isolation as risk factors for mortality: A meta-analytic review. *Perspect. Psychol. Sci.* **10**, 227–237 (2015). 25910392 10.1177/1745691614568352

[3] Leigh-Hunt, N. et al. An overview of systematic reviews on the public health consequences of social isolation and loneliness. *Public Health* **152**, 157–171. 10.1016/j.puhe.2017.07.035 (2017). 28915435 10.1016/j.puhe.2017.07.035

[4] Rafnsson, S. B., Orrell, M., d’Orsi, E., Hogervorst, E. & Steptoe, A. Loneliness, social integration, and incident dementia over 6 years: Prospective findings from the english longitudinal study of ageing. *J. Gerontol. B Psychol. Sci. Soc. Sci.* **75**, 114–124. 10.1093/geronb/gbx087 (2020). 28658937 10.1093/geronb/gbx087 PMC6909434

[5] Sonderby, L. C. & Wagoner, B. Loneliness: An integrative approach. *J. Integr. Soc. Sci.* **3**, 1–29 (2013).

[6] Perlman, D. & Peplau, L. A. *Personal Relationships in Disorder* 31–56 (Academic Press, 1981).

[7] McHugh, J., Kenny, R. A., Lawlor, B. A., Steptoe, A. & Kee, F. The discrepancy between social isolation and loneliness as a clinically meaningful metric: Findings from the Irish and English longitudinal studies of ageing (TILDA and ELSA). *Int. J. Geriatr. Psychiatry* **32**, 664–674 (2017). 27246181 10.1002/gps.4509

[8] Berkman, L. F. & Syme, S. L. Social networks, host resistance, and mortality: A nine-year follow-up study of Alameda County residents. *Am. J. Epidemiol.* **109**, 186–204 (1979). 425958 10.1093/oxfordjournals.aje.a112674

[9] Ernst, M. *Einsamkeit–Modelle, Ursachen, Interventionen*. (utb GmbH, 2024).

[10] Buecker, S., Denissen, J. J. & Luhmann, M. A propensity-score matched study of changes in loneliness surrounding major life events. *J. Pers. Soc. Psychol.* **121**, 669 (2021). 33119390 10.1037/pspp0000373

[11] Long, E. et al. COVID-19 pandemic and its impact on social relationships and health. *J. Epidemiol. Commun. Health* **76**, 128–132. 10.1136/jech-2021-216690 (2022). 10.1136/jech-2021-216690 PMC8380476 34413184

[12] Ferreira, J. M., Merçon-Vargas, E. A. & Midgette, A. J. Sociability, social isolation, and social interaction during the first months of COVID-19 pandemic: A qualitative analysis of Brazilian, Finnish, and American adults. *Trends Psychol.*10.1007/s43076-022-00172-9 (2022). 10.1007/s43076-022-00172-9 PMC8942390 40479170

[13] Schachter, S. *The Psychology of Affiliation: Experimental Studies of the Sources of Gregariousness* (Stanford University Press, 1959).

[14] Gruber, J. et al. Mental health and clinical psychological science in the time of COVID-19: Challenges, opportunities, and a call to action. *Am. Psychol.* **76**, 409–426. 10.1037/amp0000707 (2021). 32772538 10.1037/amp0000707 PMC7873160

[15] Salari, N. et al. Prevalence of stress, anxiety, depression among the general population during the COVID-19 pandemic: A systematic review and meta-analysis. *Global Health* **16**, 57. 10.1186/s12992-020-00589-w (2020). 32631403 10.1186/s12992-020-00589-w PMC7338126

[16] McPherson, K. E., McAloney-Kocaman, K., McGlinchey, E., Faeth, P. & Armour, C. Longitudinal analysis of the UK COVID-19 psychological wellbeing study: Trajectories of anxiety, depression and COVID-19-related stress symptomology. *Psychiatry Res.* **304**, 114138. 10.1016/j.psychres.2021.114138 (2021). 34388511 10.1016/j.psychres.2021.114138 PMC8424320

[17] Pierce, M. et al. Mental health before and during the COVID-19 pandemic: A longitudinal probability sample survey of the UK population. *Lancet Psychiatry* **7**, 883–892. 10.1016/S2215-0366(20)30308-4 (2020). 32707037 10.1016/S2215-0366(20)30308-4 PMC7373389

[18] Hajek, A. & Konig, H. H. Which factors contribute to loneliness among older Europeans? Findings from the survey of health, ageing and retirement in Europe: Determinants of loneliness. *Arch. Gerontol. Geriatr.* **89**, 104080. 10.1016/j.archger.2020.104080 (2020). 32371343 10.1016/j.archger.2020.104080

[19] Beutel, M. E. et al. Emotional and tangible social support in a German population-based sample: Development and validation of the brief social support scale (BS6). *PLoS ONE* **12**, e0186516. 10.1371/journal.pone.0186516 (2017). 29023540 10.1371/journal.pone.0186516 PMC5638535

[20] Ernst, M., Brähler, E., Kruse, J., Kampling, H. & Beutel, M. E. Does loneliness lie within? Personality functioning shapes loneliness and mental distress in a representative population sample. *J. Affect. Disord. Rep.*10.1016/j.jadr.2023.100486 (2023).

[21] Dykstra, P. A. & Fokkema, T. Social and emotional loneliness among divorced and married men and women: Comparing the deficit and cognitive perspectives. *Basic Appl. Soc. Psychol.* **29**, 1–12 (2007).

[22] Heu, L. C., van Zomeren, M. & Hansen, N. Lonely alone or lonely together? A cultural-psychological examination of individualism–collectivism and loneliness in five European countries. *Pers. Soc. Psychol. Bull.* **45**, 780–793. 10.1177/0146167218796793 (2019). 30264659 10.1177/0146167218796793 PMC6449799

[23] Varga, T. V. et al. Loneliness, worries, anxiety, and precautionary behaviours in response to the COVID-19 pandemic: A longitudinal analysis of 200,000 Western and Northern Europeans. *Lancet Reg. Health Eur.* **2**, 100020. 10.1016/j.lanepe.2020.100020 (2021). 33870246 10.1016/j.lanepe.2020.100020 PMC8042675

[24] Bu, F., Steptoe, A. & Fancourt, D. Who is lonely in lockdown? Cross-cohort analyses of predictors of loneliness before and during the COVID-19 pandemic. *Public Health* **186**, 31–34. 10.1016/j.puhe.2020.06.036 (2020). 32768621 10.1016/j.puhe.2020.06.036 PMC7405905

[25] Bu, F., Steptoe, A. & Fancourt, D. Loneliness during a strict lockdown: Trajectories and predictors during the COVID-19 pandemic in 38,217 United Kingdom adults. *Soc. Sci. Med.* **265**, 113521. 10.1016/j.socscimed.2020.113521 (2020). 33257177 10.1016/j.socscimed.2020.113521 PMC7768183

[26] Luchetti, M. et al. The trajectory of loneliness in response to COVID-19. *Am Psychol* **75**, 897–908. 10.1037/amp0000690 (2020). 32567879 10.1037/amp0000690 PMC7890217

[27] Health, N. I. O. *NIH Style Guide - Age*, <https://www.nih.gov/nih-style-guide/age> (2025).

[28] Berg-Weger, M. & Morley, J. E. Loneliness in old age: An unaddressed health problem. *J. Nutr. Health Aging* **24**, 243–245. 10.1080/03601277.2017.1420005 (2020). 32115602 10.1007/s12603-020-1323-6 PMC7223173

[29] Koch-Institut, R. *Bundesweite Studie zur Gesundheit älterer Menschen in Deutschland. Wie geht es den Menschen ab 65 Jahren?* (RKI, 2023).

[30] Dahlberg, L. Loneliness during the COVID-19 pandemic. *Aging Ment. Health* **25**, 1161–1164. 10.1080/13607863.2021.1875195 (2021). 33491474 10.1080/13607863.2021.1875195

[31] Emerson, K. G. Coping with being cooped up: Social distancing during COVID-19 among 60+ in the United States. *Rev. Panam. Salud. Publica* **44**, e81. 10.26633/RPSP.2020.81 (2020). 32612645 10.26633/RPSP.2020.81 PMC7323755

[32] van Tilburg, T. G., Steinmetz, S., Stolte, E., van der Roest, H. & de Vries, D. H. Loneliness and mental health during the COVID-19 pandemic: A study among dutch older adults. *J. Gerontol. B Psychol. Sci. Soc. Sci.* **76**, e249–e255. 10.1093/geronb/gbaa111 (2021). 32756931 10.1093/geronb/gbaa111 PMC7454922

[33] Fuller, H. R. & Huseth-Zosel, A. Older adults’ loneliness in early COVID-19 social distancing: Implications of rurality. *J. Gerontol. B Psychol. Sci. Soc. Sci.* **77**, e100–e105. 10.1093/geronb/gbab053 (2022). 33928371 10.1093/geronb/gbab053 PMC8135449

[34] Kivi, M., Hansson, I. & Bjalkebring, P. Up and about: Older adults’ well-being during the COVID-19 pandemic in a swedish longitudinal study. *J. Gerontol. B Psychol. Sci. Soc. Sci.* **76**, e4–e9. 10.1093/geronb/gbaa084 (2021). 32599622 10.1093/geronb/gbaa084 PMC7337833

[35] Lin, T. et al. Loneliness progression among older adults during the early phase of the COVID-19 pandemic in the United States and Canada. *J. Gerontol. B Psychol. Sci. Soc. Sci.* **77**, e23–e29. 10.1093/geronb/gbab229 (2022). 34905015 10.1093/geronb/gbab229 PMC8974322

[36] Mayerl, H., Stolz, E. & Freidl, W. Trajectories of loneliness, depressive symptoms, and anxiety symptoms during the COVID-19 pandemic in Austria. *Public Health* **212**, 10–13. 10.1016/j.puhe.2022.08.004 (2022). 36174437 10.1016/j.puhe.2022.08.004 PMC9411147

[37] van der Velden, P. G. et al. Anxiety and depression symptoms, the recovery from symptoms, and loneliness before and after the COVID-19 outbreak among the general population: Findings from a Dutch population-based longitudinal study. *PLoS ONE* **16**, e0245057. 10.1371/journal.pone.0245057 (2021). 33411843 10.1371/journal.pone.0245057 PMC7790276

[38] Bartres-Faz, D. et al. The paradoxical effect of COVID-19 outbreak on loneliness. *BJPsych Open* **7**, e30. 10.1192/bjo.2020.163 (2021). 33427159 10.1192/bjo.2020.163 PMC7804076

[39] Landmann, H. & Rohmann, A. When loneliness dimensions drift apart: Emotional, social and physical loneliness during the COVID-19 lockdown and its associations with age, personality, stress and well-being. *Int. J. Psychol.* **57**, 63–72. 10.1002/ijop.12772 (2022). 33973238 10.1002/ijop.12772 PMC8239761

[40] Dynamore. YNACORE-L(DE) Längsschnittliche DynaMORE Studie zu psychologischen Reaktionen auf die COVID-19-Pandemie.(2020).

[41] DIW Berlin / SOEP (Ed.): SOEP-Core-2020: Corona round 2 (with reference to variables), SOEP Survey Papers, No. 1248, Deutsches Institut für Wirtschaftsforschung (DIW), Berlin. (2023).

[42] Polenick, C. A. et al. Loneliness during the COVID-19 pandemic among older adults with chronic conditions. *J. Appl. Gerontol.* **40**, 804–813 (2021). 33641513 10.1177/0733464821996527 PMC8238795

[43] Zhang, Y. & Ma, Z. F. Impact of the COVID-19 pandemic on mental health and quality of life among local residents in Liaoning Province, China: A cross-sectional study. *Int. J. Environ. Res. Public Health* **17**, 2381 (2020). 32244498 10.3390/ijerph17072381 PMC7177660

[44] Kadowaki, L. & Wister, A. Older adults and social isolation and loneliness during the COVID-19 pandemic: an integrated review of patterns, effects, and interventions. *Can. J. Aging/La Revue canadienne du vieillissement* **42**, 199–216 (2023). 10.1017/S0714980822000459 36345649

[45] Gardiner, C., Geldenhuys, G. & Gott, M. Interventions to reduce social isolation and loneliness among older people: An integrative review. *Health Soc. Care Commun.* **26**, 147–157 (2018). 10.1111/hsc.12367 27413007

[46] Goll, J. C., Charlesworth, G., Scior, K. & Stott, J. Barriers to social participation among lonely older adults: The influence of social fears and identity. *PLoS ONE* **10**, e0116664 (2015). 25706933 10.1371/journal.pone.0116664 PMC4338142

[47] Masi, C. M., Chen, H.-Y., Hawkley, L. C. & Cacioppo, J. T. A meta-analysis of interventions to reduce loneliness. *Pers. Soc. Psychol. Rev.* **15**, 219–266 (2011). 20716644 10.1177/1088868310377394 PMC3865701

[48] Saito, T., Kai, I. & Takizawa, A. Effects of a program to prevent social isolation on loneliness, depression, and subjective well-being of older adults: A randomized trial among older migrants in Japan. *Arch. Gerontol. Geriatr.* **55**, 539–547 (2012). 22564362 10.1016/j.archger.2012.04.002

[49] Zagic, D., Wuthrich, V. M., Rapee, R. M. & Wolters, N. Interventions to improve social connections: a systematic review and meta-analysis. *Soc. Psychiatry Psychiatric Epidemiol.* **57**, 885–906 (2021). 10.1007/s00127-021-02191-w 34796368

[50] Wild, P. S. et al. The Gutenberg Health Study. *Bundesgesundheitsblatt Gesundheitsforschung Gesundheitsschutz* **55**, 824–829. 10.1007/s00103-012-1502-7 (2012). 22736163 10.1007/s00103-012-1502-7

[51] Petersen, J. et al. The burdens of poverty during the COVID-19 pandemic. *Front. Sociol.* **7**, 995318. 10.3389/fsoc.2022.995318 (2022). 36505762 10.3389/fsoc.2022.995318 PMC9731111

[52] Reinwarth, A. C., Ernst, M., Krakau, L., Brahler, E. & Beutel, M. E. Screening for loneliness in representative population samples: Validation of a single-item measure. *PLoS ONE* **18**, e0279701. 10.1371/journal.pone.0279701 (2023). 36928277 10.1371/journal.pone.0279701 PMC10019616

[53] Beutel, M. E. et al. Loneliness in the general population: prevalence, determinants and relations to mental health. *BMC Psychiatry* **17**, 97. 10.1186/s12888-017-1262-x (2017). 28320380 10.1186/s12888-017-1262-x PMC5359916

[54] Ernst, M. et al. Loneliness predicts suicidal ideation and anxiety symptoms in long-term childhood cancer survivors. *Int. J. Clin. Health Psychol.* **21**, 100201. 10.1016/j.ijchp.2020.10.001 (2021). 33363584 10.1016/j.ijchp.2020.10.001 PMC7753031

[55] Reinwarth, A. C. et al. Loneliness and social anxiety in the general population over time–results of a cross-lagged panel analysis. *Psychol. Med.* **54**, 4551–4560 (2024). 10.1017/S0033291724001818 39726175

[56] Richter, D. & Weinhardt, M. *Psychologische und Sozialwissenschaftliche Kurzskalen: Standardisierte Erhebungsinstrumente für Wissenschaft und Praxis* 1st edn. (Mwv Medizinisch Wissenschaftliche Verlagsges, 2013).

[57] Kocalevent, R.-D., Zenger, M., Hinz, A., Klapp, B. & Brähler, E. Resilient coping in the general population: Standardization of the brief resilient coping scale (BRCS). *Health Qual. Life Outcomes* **15**, 251. 10.1186/s12955-017-0822-6 (2017). 29282066 10.1186/s12955-017-0822-6 PMC5746021

[58] Kocalevent, R. D., Hinz, A. & Brahler, E. Standardization of the depression screener patient health questionnaire (PHQ-9) in the general population. *Gen. Hosp. Psychiatry* **35**, 551–555. 10.1016/j.genhosppsych.2013.04.006 (2013). 23664569 10.1016/j.genhosppsych.2013.04.006

[59] Löwe, B. et al. A 4-item measure of depression and anxiety: valid$ation and standardization of the Patient Health Questionnaire-4 (PHQ-4) in the general population. *J. Affect. Disord.* **122**, 86–95. 10.1016/j.jad.2009.06.019 (2010). 19616305 10.1016/j.jad.2009.06.019

[60] Wiltink, J. et al. Mini-social phobia inventory (mini-SPIN): Psychometric properties and population based norms of the German version. *BMC Psychiatry* **17**, 377. 10.1186/s12888-017-1545-2 (2017). 29178857 10.1186/s12888-017-1545-2 PMC5702247

